# Effect of different intravenous iron preparations on lymphocyte intracellular reactive oxygen species generation and subpopulation survival

**DOI:** 10.1186/1471-2369-11-16

**Published:** 2010-08-17

**Authors:** Ajay Gupta, Jiaying Zhuo, Junli Zha, Srinivasa Reddy, Jonathan Olp, Amy Pai

**Affiliations:** 1 Rockwell Medical, Wixom, MI, USA; 2 UCLA and Charles Drew University Schools of Medicine, Los Angeles, CA, USA; 3 Department of Medicine, University of California, Los Angeles, CA 90095, USA; 4 Molecular and Medical Pharmacology, University of California, Los Angeles, CA 90095, USA; 5 University of New Mexico, Albuquerque, NM, USA; 6 ANephRx Albany Nephrology Pharmacy Group, Albany College of Pharmacy and Health Sciences, Albany, NY, USA

## Abstract

**Background:**

Infections in hemodialysis (HD) patients lead to high morbidity and mortality rates and are associated with early cardiovascular mortality, possibly related to chronic inflammation. Intravenous (IV) iron is widely administered to HD patients and has been associated with increased oxidative stress and dysfunctional cellular immunity. The purpose of this study was to examine the effect of three commercially available IV iron preparations on intracellular reactive oxygen species generation and lymphocyte subpopulation survival.

**Methods:**

Peripheral blood mononuclear cells (PBMC) were isolated from healthy donor buffy coat. PBMC were cultured and incubated with 100 μg/mL of sodium ferric gluconate (SFG), iron sucrose (IS) or iron dextran (ID) for 24 hours. Cells were then probed for reactive oxygen species (ROS) with dichlorofluorescein-diacetate. In separate studies, isolated PBMCs were incubated with the 25, 50 or 100 μg/mL iron concentrations for 72 hours and then stained with fluorescein conjugated monoclonal antibodies for lymphocyte subpopulation identification. Untreated PBMCs at 24 hours and 72 hours served as controls for each experiment.

**Results:**

All three IV iron preparations induced time dependent increases in intracellular ROS with SFG and IS having a greater maximal effect than ID. The CD4+ lymphocytes were most affected by IV iron exposure, with statistically significant reduction in survival after incubation with all three doses (10, 25 and 100 μg/mL) of SFG, IS and ID.

**Conclusion:**

These data indicate IV iron products induce differential deleterious effects on CD4+ and CD16+ human lymphocytes cell populations that may be mediated by intracellular reactive oxygen species generation. Further studies are warranted to determine the potential clinical relevance of these findings.

## Background

Infection is the second leading cause of mortality among hemodialysis (HD) patients. Infections in HD patients are also associated with increased cardiovascular mortality, which may be related to immune system dysfunction resulting in recurrent infections that contribute to chronic inflammation and accelerated atherosclerosis [1]. Cellular dysfunction of both innate (e.g. T cells and macrophages) and adaptive (e.g. B cells) immunity is well described in patients with chronic kidney disease (CKD) and may be attributable to many factors including accumulated uremic toxin burden, bio-incompatible dialysis membranes, anemia, malnutrition and altered iron metabolism [1,2].

Transfusional iron overload has long been linked with immune dysfunction including defective neutrophil and macrophage chemotaxis and phagocytic activity as well as decreased natural killer (NK) cell activity [3]. Intravenous (IV) iron compounds, including iron sucrose and iron dextran, which are widely administered to HD patients, have been associated with depressed neutrophil intracellular killing capacity, reduced polymorphonuclear cell hydrogen peroxide production and impaired phagocytic activity [4,5]. More recently, HD patients were noted to have T cell and NK cell proliferation defects compared to normal control populations; however, potential relationships to IV iron administration were not explored in these studies [6,7]. Poor immune response to vaccination corroborates the theory of T cell dysfunction in HD patients. IV iron administration was reported to be associated with lower post-vaccination HBsAg titers compared to patients not exposed to IV iron [8]. Clearly, more data are needed to understand the impact of IV iron on immune function in HD patients who are at high risk of infections with subsequent poor outcomes. There are currently several commercially available IV iron products in the United States, including iron dextran (INFeD^®^), sodium ferric gluconate (Ferrlecit^®^), and iron sucrose (Venofer^®^). Differences exist between the compounds with regard to molecular weight of the iron-carbohydrate group, stability of the iron-carbohydrate complex, and the rapidity with which iron is released from the iron-carbohydrate complex[9]. We have previously shown that the smaller molecular weight iron-carbohydrate complexes (sodium ferric gluconate and iron sucrose) are associated with greater appearance of free, or non-transferrin-bound, iron *in vivo *when compared to the larger molecular weight iron-carbohydrate complex, iron dextran [10].

There have been no comparative studies examining the effect of IV iron exposure on lymphocyte proliferation and subpopulation survival. The purpose of this translational *in vitro *study was to examine the effects of the three commercially available IV iron compounds on intracellular oxidative stress and survival of human lymphocytes.

## Methods

### PBMC Isolation

Peripheral blood mononuclear cells (PBMCs) from healthy blood donor buffy coat were separated from 10 mL of whole blood on Ficoll-Hypaque density gradients, washed twice in phosphate buffered saline (PBS) (pH 7.4), and centrifuged again at 1500 × g and 4°C. Isolated PBMCs were cultured in RPMI-1640 medium supplemented with 10% heat inactivated human AB serum (Mediatech Inc, Manassas VA), 100 U/mL penicillin, 100 μg/mL streptomycin, and 2 mM L-glutamine (Omega Scientific Inc, Tarzana CA) at 37°C at 5% CO_2 _(Excella Eco-170, New Brunswick Scientific, Edison, NJ).

### Reactive Oxygen Species (ROS) Detection

Intracellular oxidative stress was measured by fluorescent probe, dichlorofluorescein. Briefly, PBMCs were isolated from healthy volunteers, plated onto 96-well plates, and treated with phytohemagglutinin (PHA). The cells were treated for 2, 4, 8 and 24 hours with 100 μg/mL concentrations of IV iron agents Iron Dextran (ID) (INFeD^®^, Watson Pharma, Inc, Morristown, NJ), Iron Sucrose (IS) (Venofer^®^, American Regent, Shirley, NY) and Sodium Ferric Gluconate (SFG) (Ferrlecit^®^, Watson Pharma, Inc, Morristown, NJ). Untreated PBMCs analyzed at 24 hours served as controls and experiments were performed in triplicate. Cells were washed with Krebs-Ringer buffer and subsequently incubated in Dulbecco's modified essential medium containing 100 μM Dichlorofluorescein-diacetate (DCFH-DA) and 1% fetal bovine serum in 5% CO_2 _and 95% air at 37°C. One hour later, DCFH-DA was removed, and cells were washed with Krebs-Ringer buffer. Fluorescence was measured in a fluorescent plate reader (Spectra Max Gemini XS, Molecular Devices Sunnyvale, CA) with the temperature maintained at 37°C.

### Lymphocyte Subpopulation Analysis

PBMCs from healthy volunteers were stimulated with IV iron agents SFG, IS, and ID for 72 hours at concentrations of 10, 25, and 100 μg/mL. The PBMCs were then stained with fluorescein conjugated monoclonal antibodies and quantified by flow cytometry (BD FACS Scan (Becton Dickinson, BD Biosciences, San Jose, CA) and CellQuest Pro Version 5.2 (Rockville, MD) to assess the proportion of T helper (CD4^+^), cytotoxic T (CD8^+^), NK (CD56^+^), macrophages (CD16^+^) and B cell (CD40^+^) subpopulations among the viable cells. Untreated PBMCs served as controls and experiments were performed in duplicate.

### Statistics

A two-way analysis of variance (ANOVA) was used to compare values among the treatments. *Post hoc *multiple-comparison test with a Bonferroni (parametric-equal variance) test was done to determine significant differences among the groups. A Student's *t*-test was performed to determine significance between individual treatments.

## Results

### Intracellular Reactive Oxygen Species Generation

**Figure 1:**
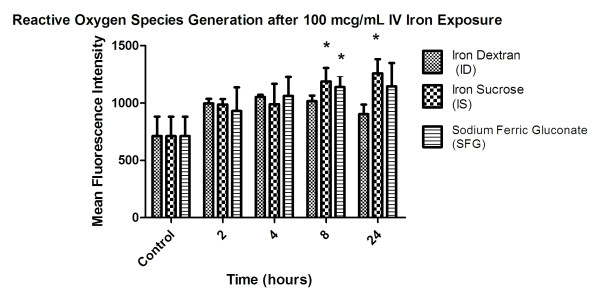
**Data are presented as mean ± SD change in MFI for iron dextran (ID), iron sucrose (IS) and sodium ferric gluconate (SFG) versus control at 2, 4, 8 and 24 hours**. *p < 0.05 vs. control. Controls represent untreated cells for each individual iron product experiments.

Incubation with 100 μg/mL IS induced significantly higher ROS generation compared to control at 8 and 24 hours. (Figure 1) Incubation with SFG induced significantly higher ROS generation at 8 hours than controls. ID treatment at all time points was not associated with any significant difference in ROS generation versus control. SFG and IS appeared to increase intracellular ROS in a time-dependent manner. At 8 hours, a 66% increase from control was measured after incubation with SFG and a 60% increase from control experiments was measured after incubation with IS. The peak mean fluorescence index (MFI) for ID, occurring at 4 hours, showed a 48% increase versus control.

### Effect of Iron on Lymphocyte Subpopulations Survival

**Figure 2:**
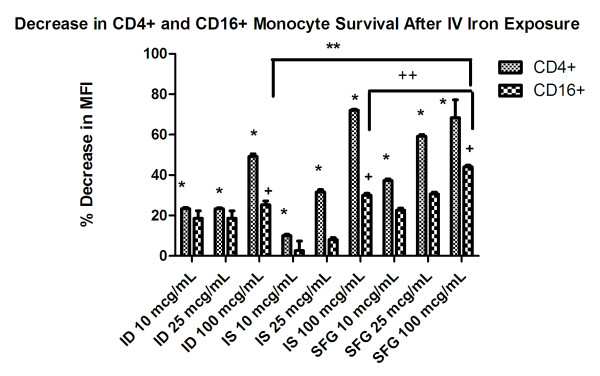
**Data are presented as mean percent reduction in MFI versus control**. For CD4+ *p < 0.05 all iron doses vs. control. For CD16+ ^+^p < 0.05 all iron products at 100 μg/mL vs. control, **ID 100 μg/mL vs. SFG 100 μg/mL, and ^++^SFG 100 μg/mL vs. IS 100 μg/mL. ID = iron dextran, IS = iron sucrose and SFG = sodium ferric gluconate.

The CD56^+^, CD40^+^, and CD8^+ ^populations were in general unaffected by IV iron preparations (data not shown). The CD16^+ ^and CD4^+ ^populations were impacted by all three IV iron preparations at all three doses (10, 25, 100 μg/mL, Figure 2). At the 10 and 25 μg/mL dose, SFG decreased the CD4^+ ^population more than the other IV iron agents (p < 0.05). However, IS at 100 μg/mL caused the largest percentage decrease in the CD4^+ ^population with a 68% reduction at 72 hours (p < 0.05). All three IV iron drug preparations decreased the CD4^+ ^population in a dose dependent manner. Survival of the CD16^+ ^population was reduced more by SFG than by ID and IS, respectively.

## Discussion

Iron is a vital metal for the proliferation and survival of all cells including those of the immune system. Iron deficiency causes a profound reduction in peripheral T cells and atrophy of the thymus. Conversely, iron overload as a result of transfusions in thalassemia is associated with decreases in circulating CD4+ T lymphocytes while expansion of CD8+CD28- T lymphocytes has been associated with iron overload in hemochromatosis. Oversaturation of transferrin above 100% and presence of non-transferrin-bound iron is associated with enhanced uptake of iron by lymphocytes and inhibition of lymphocyte proliferation [11]. *In vitro *exposure of PBMC to ferric citrate has been shown to specifically inhibit E-rosette formation, a marker of T cell presence and CD2 expression [12].

IV iron products in the form of iron-carbohydrate complexes are routinely used in HD patients and are associated with the presence of free, or non-transferrin-bound, iron. Furthermore, infection, especially related to vascular access, continues to be a monumental problem in HD patients and is associated with high morbidity and mortality rates, costly hospitalizations and treatments, and has recently been associated with cardiovascular disease [13]. Patients with chronic kidney disease are immunosuppressed and the etiology of impaired immunity is likely multifactorial possibly related to depletion of cell subsets as well as dysfunctional cell function (e.g. chemotaxis). IV iron is widely administered to HD patients and has been shown in several previous studies to adversely affect immune cell proliferation and function[4,5]. This study is the first, to our knowledge, that compares the effects of three commercially available IV iron preparations on intracellular immune cell ROS generation and survival.

This study indicates that all of the IV iron preparations are associated with increased intracellular ROS generation and reduced lymphocyte survival particularly of the CD4^+ ^and CD16^+ ^cell subpopulations. Interestingly, the smaller molecular weight IV iron compounds induced greater intracellular ROS generation, consistent with previous data regarding the appearance of non-transferrin-bound iron, which is the inciting factor driving the Fenton-Haber Weiss reaction [9,10]. These data imply that immune cell oxidative stress and reduced proliferation may be related to the molecular weight and stability of the IV iron products. Intracellular oxidative stress peaked between 8 and 24 hours which suggests that there is a lag time between administration of IV iron and potential toxicity. Thus, understanding the kinetic profile of intracellular ROS generation is important when examining these interactions *in vivo*[14].

Increased intracellular oxidative stress may predispose cells including lymphocytes to membrane destabilization (e.g. lysosomal and mitochondrial) and subsequent apoptosis. Tenopoulou *et al*. studied Jurkat cells, an immortalized T lymphocyte cell line, under conditions of continuous intracellular exposure to hydrogen peroxide [15]. A series of well-designed experiments examining the effects of iron compartmentalization, demonstrated that redox active iron released from unstable lysosomes resulted in nuclear DNA damage, mitochondrial membrane potential instability, and apoptosis. These effects were not observed when cells were incubated with the iron chelator, desferoxamine, which has been shown to be endocytosed and reach the lysosomal compartment. Studies examining the relationship between IV iron administration and alteration of intracellular iron metabolism are limited. However, Zager *et al*. observed gross uptake of iron after human proximal tubule cell incubation with iron sucrose and sodium ferric gluconate but this effect was not observed with iron dextran [16]. Intracellular iron incorporation with each IV iron compound was also confirmed by electron microscopy. Thus, it is biologically plausible that IV iron may alter the intracellular labile iron pool and H_2_O_2_-catabolizing intracellular antioxidant systems (e.g. glutathione reductase and catalase) may become overwhelmed leading to lysosomal and mitochondrial membrane destabilization and ultimately perpetuating the deleterious effects of redox-iron resulting in apoptosis.

This study demonstrated that IV iron preparations inhibit the survival of the immune cell subpopulations CD4^+ ^and CD16^+^. The SFG and IS IV iron preparations were the most injurious to the CD4^+ ^and the CD16^+ ^cell subpopulations. It has been shown that the activated lymphocytes are more susceptible to oxidative stress mediated apoptosis compared to control populations [17]. This may offer some insight to explain the reduced populations of some immune cell subsets because the immune system in HD patients can be considered to be constantly "primed" due to interaction with the dialysis membrane and possibly other inciting factors including reuse solvents [18]. A limitation to this study is the use of PBMCs from healthy subjects. Our group and others have shown that hemodialysis patients with inflammation generate more oxidative stress biomarkers acutely after IV iron administration[19,20]. Thus, it could be speculated that lymphocyte survival after iron-induced stress would be poorer in PBMCs from uremic hemodialysis patients. It could also be speculated that eliminating the CD4^+ ^subpopulation could cause the immune system to become major histocompatibility complex (MHC) class I restricted. Exogenous antigens like bacterial cell wall components are produced outside of a cell and enter the antigen presenting cells by either endocytosis or phagocytosis. MHC class II molecules are expressed on professional antigen presenting cells and present the exogenous antigen to the CD4^+ ^population. If the CD4^+ ^cell subpopulation were greatly inhibited, the processing of exogenous antigen presentation through the MHC class II pathway could also be inhibited. CD16+ monocytes are pro-inflammatory and are associated with endothelial dysfunction. Transient modest reductions in CD16+ monocyte counts, similar to the observed reductions in this *in vitro *study, have been associated with worse outcomes for cardiovascular endpoints in HD patients[21].

## Conclusion

Overall, the smaller molecular weight IV iron-carbohydrate complexes produced greater inhibition of some components of the innate immune system. These data support the development of further studies examining the effects of IV iron preparations on the effects of lymphocyte subpopulation function and survival as well as the interaction between immune dysfunction and susceptibility to certain common pathogens such as *Staphylococcal spp*. in chronic HD patients.

## Competing Interests

Dr. Gupta is Chief Scientific Officer at Rockwell Medical that is developing soluble ferric pyrophosphate for treatment of iron deficiency in end-stage renal disease. The other authors have no other conflicts of interest to disclose.

## Authors' contributions

SR, JZ and JZ carried out the PBMC isolation and flow cytometry and helped draft the manuscript. ABP and JO participated in the design of the study and performed the statistical analysis and helped to draft the manuscript. AG conceived of the study, and participated in its design and coordination and drafted the manuscript. All authors read and approved the final manuscript.

## Pre-publication history

The pre-publication history for this paper can be accessed here:

http://www.biomedcentral.com/1471-2369/11/16/prepub
